# The Changing Effect of Economic Development on the Consumption-Based Carbon Intensity of Well-Being, 1990–2008

**DOI:** 10.1371/journal.pone.0123920

**Published:** 2015-05-06

**Authors:** Andrew K. Jorgenson, Jennifer Givens

**Affiliations:** 1 Department of Sociology and Environmental Studies Program, Boston College, Chestnut Hill, Massachusetts, United States of America; 2 Department of Sociology, Washington State University, Pullman, Washington, United States of America; East China University of Science and Technology, CHINA

## Abstract

Recent sustainability science research focuses on tradeoffs between human well-being and stress placed on the environment from fossil fuel consumption, a relationship known as the carbon intensity of well-being (CIWB). In this study we assess how the effect of economic development on consumption-based CIWB—a ratio of consumption-based carbon dioxide emissions to average life expectancy—changed from 1990 to 2008 for 69 nations throughout the world. We examine the effect of development on consumption-based CIWB for the overall sample as well as for smaller samples restricted to mostly high-income OECD nations, Non-OECD nations, and more nuanced regional samples of Non-OECD nations in Africa, Asia, and Latin America. We find that the effect of economic development on CIWB increased through time for the overall sample. However, analyses of the Non-OECD and OECD samples indicate that while the effect of development on CIWB increased from null to a moderate level for the Non-OECD nations, the effect of economic development was much larger, relatively stable through time, and more unsustainable for the OECD nations. Additional findings reveal important regional differences for Non-OECD nations. In the early 1990s, increased development led to a reduction in CIWB for Non-OECD nations in Africa, but in more recent years the relationship changed, becoming less sustainable. For the samples of Non-OECD nations in Asia and Latin America, we find that economic development increased consumption-based CIWB, and increasingly so throughout the 19 year period of study.

## Introduction

A growing body of research focuses on the environmental impacts of human well-being, and the extent to which socio-economic processes and conditions influence well-being and environmental interrelationships [1–6]. Within this work, increasing attention is paid to the amount of fossil fuel energy used—and thus the amount of carbon emissions generated—to maintain a certain level of human well-being. The relationship between carbon emissions and human well-being is referred to by some scholars as the carbon intensity of well-being (CIWB) [7], an approach that builds on the broader ecological intensity of well-being framework in structural human ecology [1, 8–10]. CIWB is typically operationalized as a ratio between per capita anthropogenic carbon emissions and an established measure of human well-being (e.g., average life expectancy) where higher values mean a larger level of carbon emissions per unit of well-being, while lower values indicate a lower level of emissions per unit of human well-being [7]. A central question in this area of research asks: if reducing CIWB is a potential pathway towards greater sustainability, how can nations throughout the world successfully achieve it [7, 11–13]?

Many researchers argue that analyses focusing on the effects of economic development on CIWB are an important place to start [8, 14]. As noted by Jorgenson [7], the reasons for this focus are at least threefold. First, the relationship between carbon emissions and economic development receives significant attention in environmental social science research, with recent studies finding notable changes in the emissions and development relationship through time [15–17]. Second, the association between human well-being and economic development is a fundamental question in public health scholarship. While most research reveals strong relationships between well-being and economic development, some studies find that the strength of the relationship modestly weakens through time [18]. Third, a focus on the effects of economic development on CIWB is consistent with the foundations of sustainability science in general, which, among other things, underscores the necessity in considering the connections between environmental conditions, societal well-being, and economic growth [19].

In this study we aim to advance the emerging body of research on development and CIWB. Rather than employing the production-based measures of carbon emissions used in past studies of CIWB [7], we instead use consumption-based estimates of carbon emissions, the focus of much global sustainability analyses [6, 13, 16, 20–24]. Prior research shows that higher-income, more-developed countries tend to be net importers of carbon emissions, so their consumption-based emissions are greater than their production-based emissions, while the reverse is generally true for lower-income, developing countries [24]. And generally speaking, higher-income, more-developed nations tend to have higher levels of human well-being than lower-income, developing nations [18, 25]. Thus, we suggest that using the consumption-based carbon emissions measures in this study of development and CIWB allows for a more accurate assessment of the carbon emissions implications of human well-being for nations, while taking into consideration the complexities of global trade and production networks [26], and what has been referred to by environmental social scientists as potential environmental load displacement [27–29]. The use of consumption-based carbon emissions in the present study of CIWB is also consistent with the general logic of prior research in the ecological intensity of well-being tradition, which oftentimes use the ecological footprint of nations, a well-known comprehensive measure of consumption-based environmental demand [1, 3, 8, 10].

Using the consumption-based emissions data, we create national-level measures of consumption-based CIWB. We employ statistical modeling techniques to assess the effect of economic development on consumption-based CIWB and how it might change through time. We analyze multiple samples of nations, including an overall sample as well as samples restricted to OECD nations, Non-OECD nations, and more nuanced, regionally-defined samples of Non-OECD nations. As shown in other studies, such an approach allows for investigating broad-based human / environment relationships as well as those potentially situated within narrower socioeconomic and regional contexts [7, 30–32]. And of particular relevance for this study, sustainability scientists argue that such regional analyses should be better integrated into future climate change mitigation assessments of the Intergovernmental Panel on Climate Change as well as other global organizations focusing on climate change mitigation and broader sustainability efforts [33–34].

## Materials and Methods

### The Datasets

We estimate the same model of the effects of economic development on consumption-based CIWB for six different samples of nations. The first is an overall sample that includes 69 nations for the 1990 to 2008 period. These 19 years are those in which at the time of the study annual data are available for the employed consumption-based carbon emissions, life expectancy, and GDP per capita data, which we describe below. We only include nations in which annual data are available for each of the three variables for the 1990 to 2008 period. Such perfectly balanced panels are ideal for this study’s research design, since interaction variables for GDP per capita and dummy variables for each yearly observation are employed to assess the extent to which the effect of economic development on CIWB might change through time [15, 30, 33, 35]. While the overall sample accounts for the majority of the world’s population, an unintended consequence of this approach is the exclusion of nations where data are not available for each year, most notably former Soviet nations and small island nations. The CIWB and GDP per capita data for the overall sample are provided in S1 Dataset.

The second and third samples split the first overall sample of nations into those that are OECD nations (25 mostly high-income nations) and those that are Non-OECD nations (44 developing nations), a practice well-established in past decades of research across various disciplines. The fourth through sixth samples split the Non-OECD nations sample into regionally-defined samples, labeled as Non-OECD Africa (16 nations), Non-OECD Asia (15 nations), and Non-OECD Latin America (13 nations). Limited degrees of freedom preclude us from creating smaller-scale regional Non-OECD samples, or regional samples of OECD nations. Table 1 lists the countries included in the analyses.

**Table 1:** Countries Included in the Analyses.

OECD	Non-OECD Africa	Non-OECD Asia	Non-OECD Latin America
Australia	Botswana	Bangladesh	Argentina
Austria	Egypt	Cambodia	Bolivia
Belgium	Ethiopia	China	Brazil
Canada	Madagascar	Hong Kong	Colombia
Chile	Malawi	India	Costa Rica
Denmark	Mauritius	Indonesia	Ecuador
Finland	Morocco	Iran	Guatemala
France	Mozambique	Lao PDR	Nicaragua
Germany	Nigeria	Malaysia	Panama
Greece	Senegal	Pakistan	Paraguay
Ireland	South Africa	Philippines	Peru
Italy	Tanzania	Singapore	Uruguay
Japan	Tunisia	Sri Lanka	Venezuela
Korea	Uganda	Thailand	
Mexico	Zambia	Vietnam	
Netherlands	Zimbabwe		
New Zealand			
Norway			
Portugal			
Spain			
Sweden			
Switzerland			
Turkey			
United Kingdom			
United States of America			

### Dependent Variable

Consistent with Jorgenson [7], in this study the carbon intensity of well-being (CIWB) is a ratio between anthropogenic carbon emissions and a measure of human well-being. In the present analyses CIWB is measured as per capita consumption-based carbon dioxide emissions divided by average life expectancy at birth. We employ the consumption-based measures of carbon emissions available from Peters et al. [24].

Peters et al. [24] use estimates of carbon dioxide emissions from production, adjusted for trade, to create measures of carbon emissions from consumption. These measures are also referred to as carbon emissions embodied in trade since they account for the emissions generated in the processes of production, which are then attributed to the country of consumption rather than production, using input-output analysis techniques. The measures include emissions from fossil fuel combustion, cement production, and gas flaring. Additional details on the methods used to create the consumption-based carbon emissions data are available in the Appendix for Peters et al. [24].

The annual consumption-based carbon emissions data are provided in millions of metric tons. Using total population size data from the World Bank’s *World Development Indicators* database (http://databank.worldbank.org/, accessed March 14, 2014), we convert the total emissions data into per capita measures (i.e., per capita emissions in metric tons), and employ the per capita measures as the numerator for the CIWB ratio.

Life expectancy at birth, the denominator for CIWB, indicates the average number of years a newborn infant would live if prevailing patterns of mortality at the time of its birth were to persist throughout its life. We obtain these data from the World Bank’s *World Development Indicators* database (http://databank.worldbank.org/, accessed March 14, 2014). Average life expectancy is well measured in most countries, it has been the focus of much demographic and public health research and data are available for a large number of nations in regions throughout the world. Life expectancy is not the only appropriate measure of human well-being, but it is well-established in various areas of research, it is a widely accepted indicator of well-being, and it is employed in recent studies of the carbon intensity and the ecological intensity of well-being [7–8, 10, 13]. Future studies of CIWB could certainly employ other objective measures of human well-being as the denominator, such as poverty rates, or measures of subjective well-being [3].

Employing a ratio as a dependent variable creates a complication that must be resolved prior to the analysis. Since the variability of the numerator and the dominator can differ substantially, a ratio can be dominated by one or the other. In the overall dataset for the current study the coefficient of variation (standard deviation / mean) for the consumption-based carbon emissions data is 1.012, and for average life expectancy the coefficient of variation is. 132. Thus the variation in carbon emissions per capita—the numerator—is notably larger than the variation in life expectancy—the dominator. Under such conditions the variation in the carbon emissions data could drive variation in the ratio.

To resolve this complication we take the same approach pioneered by Dietz et al. [8] and later used by others [7, 10, 14]. We constrain the coefficient of variation of the numerator and denominator to be equal by adding a constant to the numerator, which shifts the mean without changing the variance. For our data the coefficients of variation for the two variables can be made equal by adding 38.0 to the per capita consumption-based emissions data. Doing so leads to the per capita carbon emissions data and the life expectancy data each having a coefficient of variation of. 132. Thus, our employed measure of the consumption-based carbon intensity of well-being is:

$$CIWB=\left[\left(ConCO_{2}PC+38.0\right)/\;LE\right]\;*100$$

where *CIWB* is the carbon intensity of well-being, *ConCO*_2_ *PC* is consumption-based carbon dioxide emissions per capita, and *LE* is average life expectancy. Consistent with past research [7–8], we multiple by 100 to scale the ratio.

### Independent Variables

We use gross domestic product (GDP) per capita to measure nations’ levels of economic development. These data are in constant 2005 U.S. dollars (adjusted for inflation), and obtained from the *World Development Indicators* database (http://databank.worldbank.org/, accessed March 14, 2014). We use interactions between GDP per capita and dummy variables for each year (1990–2008), with 1990 as the reference year, which allow us to assess the extent to which the effect of economic development on consumption-based CIWB increases or decreases through time [35].

### Model Estimation Technique

We use a time-series cross-sectional Prais-Winsten regression model with panel-corrected standard errors (PCSE), allowing for disturbances that are heteroskedastic and contemporaneously correlated across panels. We employ PCSE because the feasible generalized least-squares estimator that is often used to analyze panel data produces standard errors that can lead to extreme overconfidence with datasets that do not have very many more time periods than panels. We correct for AR(1) disturbances (first-order autocorrelation) within panels, and since we have no theoretical basis for assuming the process is panel specific, we treat the AR(1) process as common to all panels [36]. We include country-specific and year-specific intercepts to control for both country-specific and year-specific effects, the equivalent of a two-way fixed effects model. We note that this modeling technique controls out between-country variation in favor of estimating within-country effects, a relatively conservative approach commonly used in panel analyses.

In line with past studies of CIWB and related topics [7–8, 13], all non-dichotomous variables are transformed with the base 10 logarithm (i.e., “log”). Thus, the regression models estimate elasticity coefficients where the coefficient for the independent variable is the estimated net percentage change in the dependent variable associated with a 1% increase in the independent variable.

We estimate the same two-way fixed effects elasticity model for the overall sample as well as each of the five reduced samples. The estimated model is as follows:

$$\begin{array}{l} CIWB_{it}\left(\text{log}\right)=\beta_{1}GDP\;per\;capita_{it}\left(\text{log}\right)+\;\beta_{2}year1991_{t}+\ldots +\beta_{19}year2008_{t}+\\ \beta_{20}GDP\;per\;capita_{it}\left(\text{log}\right)*year1991_{t}+\;\ldots \;+\;\beta_{37}GDP\;per\;capita_{it}\left(\text{log}\right)*\\ year2008_{t}\;+\;u_{i}+e_{it} \end{array}$$

Where the dependent variable, *CIWB*_*it*_, is the consumption-based carbon intensity of well-being, and the model includes GDP per capita (*β*_1_ *GDP per capita*_*it*_), the year-specific intercepts β_6_year1965_t_ + … + β_14_year2005_t_), the interactions between GDP per capita and the dummy variables for each year (*β*_20_ *GDP per capita*_*it*_ * *year*1991_*t*_ + … + *β*_37_ *GDP per capita*_*it*_ * *year*2008_*t*_) where 1990 is the reference category, the country-specific intercepts (*u*_*i*_), and the disturbance term unique to each country at each point in time (*e*_*it*_). As a reminder, all non-dichotomous variables are transformed with the base 10 logarithm (log). The coefficient for GDP per capita is the unit change in the dependent variable for each unit increase in GDP per capita and captures the effect for the base year, 1990. The overall effect of GDP per capita for the other time points (i.e., 1 991, 1992, … 2007, 2008) equals the sum of the coefficient for GDP per capita and the appropriate interaction term if the latter is statistically significant [35]. This approach allows the effect of log GDP per capita to vary across years.

The use of interactions between GDP per capita and time dummy variables is the same modeling approach employed in Jorgenson’s [7] longitudinal study of production-based CIWB. However, in this study of consumption-based CIWB, we have annual observations from 1990 to 2008 (due to data availability as described above) and thus interactions between annual time dummy variables and GDP per capita, while Jorgenson’s [7] study includes observations and interaction variables in five year increments, but for a much wider time period (i.e., 1970 to 2009).

## Results and Discussion

Table 2 reports the full findings for the estimated model for each of the six samples of nations. The close to perfect R-square statistics are largely due to the unreported country-specific and year-specific intercepts (the two-way fixed effects). The two-way fixed effects help account for omitted variable bias, which would be of greater concern if the fixed effects were excluded since the estimated models do not include non-dichotomous predictors other than GDP per capita that are known to impact both carbon emissions and average life expectancy. Table 3 provides the elasticity coefficients for the estimated effects of GDP per capita for each year, which are based on the tests of statistical significance reported in Table 2 for the interactions between time and GDP per capita. Thorough sensitivity analyses indicate that none of the six samples include any overly influential cases.

**Table 2:** The Effects of GDP Per Capita on Consumption-Based CIWB, 1990–2008: Elasticity Coefficients from Two-Way Fixed Effects Prais-Winston Regression Models.

						Non-OECD
			Combined	Non-OECD	Non-OECD	Latin
	All	OECD	Non-OECD	Africa	Asia	America
GDP per capita	.045**	.137***	-.012	-.078**	-.026	.013
	(.0169)	(.0194)	(.0172)	(.0276)	(.0202)	(.0201)
GDP per capita X 1991	.001***	.005***	.001**	.002***	.002***	.006***
	(.0001)	(.0009)	(.0004)	(.0006)	(.0002)	(.0013)
GDP per capita X 1992	-.001	.004**	.003***	.006***	.006***	.007***
	(.0019)	(.0013)	(.0006)	(.0012)	(.0002)	(.0021)
GDP per capita X 1993	.002***	.002	.023***	.008***	.053***	.011***
	(.0002)	(.0016)	(.0007)	(.0010)	(.0003)	(.0023)
GDP per capita X 1994	.002***	.003*	.027***	.012***	.062***	.014***
	(.0002)	(.0015)	(.0008)	(.0014)	(.0004)	(.0024)
GDP per capita X 1995	.002***	.002	.027***	.014***	.060***	.017***
	(.0002)	(.0018)	(.0008)	(.0012)	(.0004)	(.0022)
GDP per capita X 1996	.003***	.009***	.026***	.019***	.057***	.019***
	(.0003)	(.0016)	(.0007)	(.0013)	(.0004)	(.0021)
GDP per capita X 1997	.003***	.004*	.031***	.024***	.064***	.024***
	(.0003)	(.0018)	(.0008)	(.0014)	(.0004)	(.0023)
GDP per capita X 1998	.003***	.016***	.025***	.030***	.046***	.031***
	(.0002)	(.0017)	(.0007)	(.0015)	(.0006)	(.0024)
GDP per capita X 1999	.003***	.004*	.029***	.037***	.053***	.031***
	(.0003)	(.0015)	(.0007)	(.0016)	(.0007)	(.0021)
GDP per capita X 2000	.005***	.002	.035***	.048***	.062***	.027***
	(.0003)	(.0020)	(.0007)	(.0017)	(.0006)	(.0020)
GDP per capita X 2001	.004***	.005***	.034***	.053***	.056***	.035***
	(.0003)	(.0014)	(.0006)	(.0017)	(.0009)	(.0019)
GDP per capita X 2002	.005***	.003*	.033***	.054***	.053***	.030***
	(.0003)	(.0014)	(.0007)	(.0019)	(.0009)	(.0018)
GDP per capita X 2003	.009***	.008***	.039***	.071***	.056***	.032***
	(.0003)	(.0015)	(.0008)	(.0020)	(.0010)	(.0019)
GDP per capita X 2004	.012***	.016***	.043***	.077***	.058***	.033***
	(.0004)	(.0017)	(.0008)	(.0019)	(.0009)	(.0018)
GDP per capita X 2005	.015***	.016***	.050***	.082***	.069***	.034***
	(.0004)	(.0019)	(.0008)	(.0019)	(.0010)	(.0020)
GDP per capita X 2006	.017***	.010***	.055***	.087***	.071***	.038***
	(.0004)	(.0020)	(.0009)	(.0019)	(.0010)	(.0023)
GDP per capita X 2007	.019***	.007***	.060***	.092***	.075***	.042***
	(.0005)	(.0022)	(.0009)	(.0019)	(.0011)	(.0026)
GDP per capita X 2008	.021***	.007**	.064***	.099***	.076***	.044***
	(.0006)	(.0023)	(.0010)	(.0021)	(.0014)	(.0028)
R-square	.998	.999	.997	.998	.997	.999
Number of Nations	69	25	44	16	15	13
Number of Observations	1311	475	836	304	285	247

Notes

***p<.001

**p<.01

*p<.05 (2-tailed tests); panel corrected standard errors in parentheses

**Table 3:** Elasticity Coefficients for the Estimated Effects of GDP per Capita on Consumption-Based CIWB, 1990–2008.

						Non-OECD
			Combined	Non-OECD	Non-OECD	Latin
Year	All	OECD	Non-OECD	Africa	Asia	America
1990	.045	.137	.000	-.078	.000	.000
1991	.046	.142	.001	-.076	.002	.006
1992	.045	.141	.003	-.072	.006	.007
1993	.047	.137	.023	-.070	.053	.011
1994	.047	.140	.027	-.066	.062	.014
1995	.047	.137	.027	-.064	.060	.017
1996	.048	.146	.026	-.059	.057	.019
1997	.048	.141	.031	-.054	.064	.024
1998	.048	.153	.025	-.048	.046	.031
1999	.048	.141	.029	-.041	.053	.031
2000	.050	.137	.035	-.030	.062	.027
2001	.049	.142	.034	-.025	.056	.035
2002	.050	.140	.033	-.024	.053	.030
2003	.054	.145	.039	-.007	.056	.032
2004	.057	.153	.043	-.001	.058	.033
2005	.060	.153	.050	.004	.069	.034
2006	.062	.147	.055	.009	.071	.038
2007	.064	.144	.060	.014	.075	.042
2008	.066	.144	.064	.021	.076	.044

For the sample of all 69 nations, the effect of GDP per capita on CIWB was positive and statistically significant in the year 1990, where a one percent increase in GDP per capita led to a. 045 percent increase in CIWB. All interactions between GDP per capita and the year dummy variables are positive and statistically significant for the overall sample, and in the year 2008, a one percent increase in GDP per capita led to a. 066 percent increase in CIWB. The increasing effect of development was less pronounced in the earlier years of the study, and became moderately more pronounced from roughly 2002 until the final year in the analysis, 2008.

Turning to the sample restricted to the 25 OECD nations, a one percent increase in GDP per capita in 1990 led to a. 137 percent increase in CIWB. While the majority of interactions between GDP per capita and the yearly dummy variables are positive and statistically significant, the effect of level of development appeared to peak in the years 1998, 2004, and 2005, where in each of these years a one per cent increase in GDP per capita led to a. 153 percent increase in consumption-based CIWB. In the year 2008, the elasticity coefficient for the effect of GDP per capita was. 144, which is modestly larger than the elasticity coefficient for GDP per capita in the year 1990. Overall, the effect of GDP per capita on CIWB for the OECD nations initially increased in the 1990s, followed by minor decreases through the year 2000, which were followed by minor increases through 2004 and 2005, and then minor decreases through 2008.

For the “combined” sample of 44 Non-OECD nations, the effect of GDP per capita on CIWB in 1990 was not statistically significant. Thus, we interpret the estimated effect of level of development for this initial year in the analysis as being zero. All interactions between GDP per capita and year are positive and significant. The elasticity coefficients for the effect of GDP per capita increased from zero in 1990 to. 001 in 1991 and. 003 in 1992, and then began to increase more notably each year through 2008, where a one percent increase in GDP per capita led to a. 064 percent increase in CIWB.

**Fig 1:**
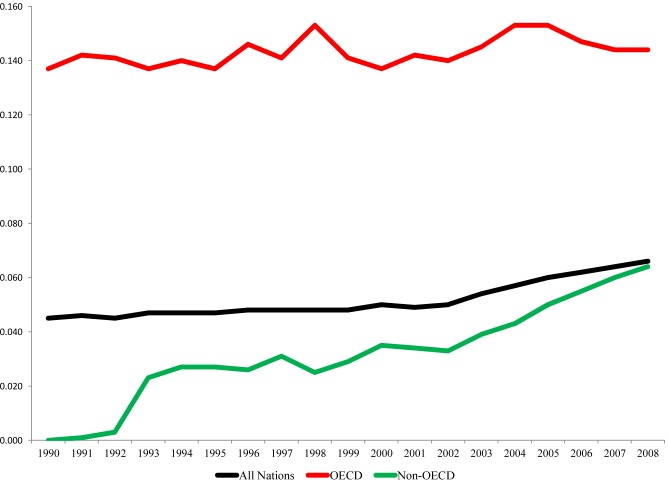
Elasticity Coefficients for the Estimated Effects of GDP per capita on Consumption-Based CIWB, 1990–2008. Coefficients are derived from the estimated models reported in Table 2.

Fig 1 graphs the annual elasticity coefficients for the sample of all 69 nations as well as the samples restricted to the 25 OECD nations and the 44 Non-OECD nations. The analyses of each of these three samples suggest unsustainable relationships between consumption-based CIWB and economic development, and the analysis of the overall sample hides notable differences in the dynamic relationships between CIWB and development for the mostly high income OECD nations relative to the Non-OECD nations. While the elasticity coefficients for the effect of GDP per capita on CIWB increased much more substantially from 1990 to 2008 for the Non-OECD nations than for the OECD nations, the coefficient of. 064 in 2008 for Non-OECD nations is notably smaller than the coefficient of. 144 for the sample of OECD nations. In other words, while development appears to have become increasingly unsustainable in Non-OECD nations, economic development continues to be much more unsustainable for the OECD nations, at least in the context of consumption-based CIWB. We now review the results of the analyses of the three samples of regionally-defined Non-OECD nations.

As noted in Table 2, for the sample of 16 Non-OECD Africa nations, in the year 1990 a one percent increase in GDP per capita led to a. 078 percent decrease in CIWB. All of the interactions between GDP per capita and the year dummy variables are positive and statistically significant, which suggests that development became increasingly less sustainable for these nations. Table 3 shows that while the elasticity coefficient for GDP per capita was still slightly negative in the year 2004 (coefficient of-.001), in the year 2005 the coefficient became positive, and suggesting that a one percent increase in GDP per capita led to a. 004 percent increase in consumption-based CIWB. The positive coefficient continued to increase, and by the year 2008 it reached a value of. 021.

Turning to the sample of 15 Non-OECD Asia nations, for the year 1990 the effect of GDP per capita on CIWB was non-significant and thus zero. All interactions between GDP per capita and year are positive and statistically significant, and with a few exceptions increase in value successively, which suggests an increasingly unsustainable relationship between consumption-based CIWB and development. By the year 2008, the elasticity coefficient for the effect of GDP per capita was. 076.

The results of the analysis of the 13 Non-OECD Latin America nations are generally consistent with the findings for the Non-OECD Asia countries. The estimated effect of GDP per capita in 1990 on CIWB for these nations was not significantly different than zero. All interactions between GDP per capita and the year dummy variables are positive and statistically significant. In 2008, a one percent increase in GDP per capita led to a. 044 percent increase in consumption-based CIWB.

**Fig 2:**
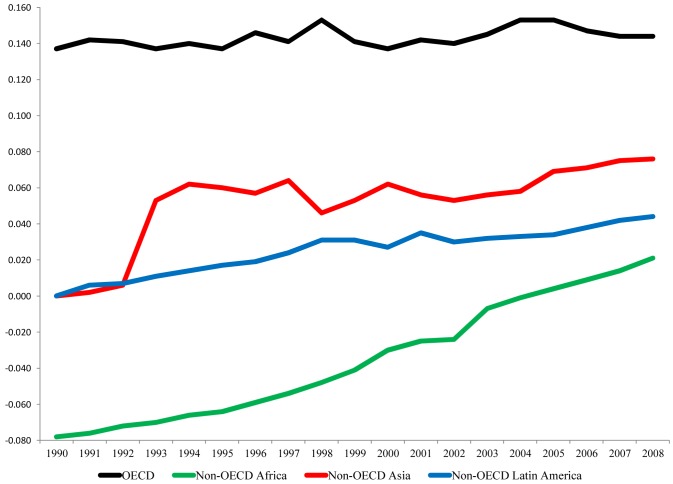
Elasticity Coefficients for the Estimated Effects of GDP per capita on Consumption-Based CIWB, 1990–2008. Coefficients are derived from the estimated models reported in Table 2.

Fig 2 graphs the annual elasticity coefficients for each of the three regionally-defined samples of Non-OECD nations as well as the sample of 25 OECD nations for comparison purposes. The plotted lines in Fig 2 underscore that the increasingly unsustainable relationship between consumption-based CIWB and development for the overall sample of Non-OECD nations is largely a function of the trajectories for the Non-OECD nations in Asia and Latin America, and to a much lesser extent the Non-OECD nations in Africa. While the latter group of nations experienced noteworthy changes in the CIWB and development relationship through time, the positive elasticity coefficient in 2008 for the effect of GDP for the Non-OECD Africa nations (.021) was less than half as large as the elasticity coefficient for the sample of Non-OECD Latin America nations (.044) and less than 1/3 the size of the elasticity coefficient for the sample of Non-OECD Asia nations (.076). As important, the elasticity coefficient for the effect of GDP per capita in the year 2008 for the sample of mostly high income OECD nations (.144) is almost twice as large as the elasticity coefficient for the sample of Non-OECD Asia nations.

To check the sensitivity of our results to the specific approach we used to measure consumption-based CIWB, we repeated our analyses using an alternative measure—the residual from regressing life expectancy on the per capita carbon emissions measures. We used the residuals from this regression as an alternative dependent variable to the CIWB ratio measure. Positive residuals indicate higher well-being relative to levels of emissions, while negative residuals indicate lower well-being relative to levels of emissions. This residual approach, which was initially used by urban political economy scholars [37–38], is used as the primary dependent variable in other studies on similar sustainability topics [3], and treated as a sensitivity analysis in past research on production-based CIWB [7]. These residuals, as expected, are close to perfectly negatively correlated with the CIWB ratio measures. The results of the sensitivity analyses are substantively identical to the reported analyses of the consumption-based CIWB ratio measure, providing added confidence in their validity.

## Conclusion

This research advances sustainability scholarship on the environmental impacts of human well-being, and the extent to which socio-economic conditions influence them [4, 7–8, 13–14, 39]. The findings highlight notable differences in the changing effect of economic development on the consumption-based carbon intensity of well-being across OECD and Non-OECD nations as well as notable differences among Non-OECD nations in different regions. The elasticity coefficients for the effect of GDP per capita on consumption-based CIWB in mostly high-income OECD nations, which remain relatively stable in value through time, are much larger in each year than for the Non-OECD nations. Further, there are important differences across the samples of Non-OECD nations in Asia, Latin America, and Africa, but all of these regions, which consist of less-developed and developing nations, experienced patterns of increasingly unsustainable relationships between CIWB and economic development during the 1990 to 2008 period of study. Thus, even with these differences in mind, this research suggests that economic development by itself is not a pathway to sustainability—at least in the context of reducing consumption-based CIWB.

The use of consumption-based carbon emissions in this study leads to differences between OECD and non-OECD nations that appear to be much more pronounced than when employing production-based, territorial measures of carbon emissions [7, 13]. In other words, studies of CIWB and other related outcomes that use measures of production-based carbon emissions might yield results that underestimate the differences between relatively higher-income and lower-income nations. As this study shows, for the sample of mostly high-income OECD nations, the effect of economic development on consumption-based CIWB is relatively time-invariant and much larger than the increasing effect of development for lower-income non-OECD nations throughout the world. Besides advancing sustainability science, the empirical identification of these Global North / Global South differences and the recognition that consumption-based measures more effectively capture societal contributions to overall anthropogenic greenhouse gas emissions [16, 20, 22, 24] could help in resolving the ongoing challenges concerning the formation and widespread acceptance of equitable climate change mitigation and adaptation international agreements [23, 40–43].

The findings for this study point to multiple avenues for future research. First, future research would do well to consider how other factors might shape the consumption-based CIWB of nations, including the presence of environmental INGOs and other sustainability-oriented civil society configurations, the regulatory mechanisms of nations and other political bodies, and the structural and spatial characteristics of populations within nations, such as urban and rural population distributions and levels of domestic income inequality. Second, as consumption-based carbon emissions data for an adequate number of nations become available for years more current than 2008, researchers should examine if the effect of development on CIWB was impacted by the recent world economic recession. Third, future research should employ qualitative comparative analysis (QCA), a technique that uses Boolean algebra and is capable of handling higher-order complex interactions between variables, to identify combinations of factors and conditions that might lead to lower levels of growth or actual reductions in CIWB (for applied examples of QCA, see [32, 44]). Analyses along these lines would help answer the call for solutions-oriented sustainability research, something that other scholars working in this particular area of scholarship have highlighted as well [11]. Lastly, with the increasing availability of comparative data at smaller scales, such as the city level [45], US state and county levels [46], and the province level in different nations [47], future research should conduct studies at such lower levels of analysis to assess the potential similarities and differences in observed relationships between environmental conditions, human well-being, and socio-economic factors at national and sub-national levels, and how the interrelationships may change through time.

## Supporting Information

S1 Dataset The Data Analyzed in the Study. (PDF)
