# Supplementary material for: The Changing Effect of Economic Development on the Consumption-Based Carbon Intensity of Well-Being, 1990–2008
Source: PLoS One. 2015 May 6;10(5):e0123920. doi: 10.1371/journal.pone.0123920 (PMC4422519; doi:10.1371/journal.pone.0123920)
Supplement: S1 Dataset — (PDF) [file pone.0123920.s001.pdf]

Country	Year	CIWB (log base 10)	GDP per capita (log base 10)
Argentina	1990	1.7632709	3.5135185
Argentina	1991	1.7638009	3.5593721
Argentina	1992	1.7637888	3.6025484
Argentina	1993	1.7613237	3.6217946
Argentina	1994	1.761641	3.6408713
Argentina	1995	1.7584465	3.6228926
Argentina	1996	1.7585883	3.6409155
Argentina	1997	1.7595165	3.669562
Argentina	1998	1.7587534	3.6809057
Argentina	1999	1.7571492	3.6611017
Argentina	2000	1.7530366	3.6530651
Argentina	2001	1.7502666	3.6291468
Argentina	2002	1.7360513	3.574944
Argentina	2003	1.7427368	3.6077813
Argentina	2004	1.744377	3.6414726
Argentina	2005	1.7447488	3.6757845
Argentina	2006	1.7467373	3.7072625
Argentina	2007	1.7491072	3.7365558
Argentina	2008	1.7481636	3.7640266
Australia	1990	1.8376384	4.3971679
Australia	1991	1.8322675	4.3901064
Australia	1992	1.8329689	4.3866926
Australia	1993	1.8310244	4.3999396
Australia	1994	1.8329267	4.4125782
Australia	1995	1.8305291	4.4243221
Australia	1996	1.8343492	4.4356228
Australia	1997	1.832102	4.4473821
Australia	1998	1.8380265	4.4620724
Australia	1999	1.8265236	4.4781111
Australia	2000	1.8196913	4.489323
Australia	2001	1.8141962	4.4916391
Australia	2002	1.8226447	4.5030086
Australia	2003	1.8220828	4.5111225
Australia	2004	1.822378	4.5237361
Australia	2005	1.8230247	4.5316288
Australia	2006	1.8220395	4.5382394
Australia	2007	1.8213357	4.5516736
Australia	2008	1.8051954	4.5590375
Austria	1990	1.8340172	4.4477846
Austria	1991	1.8351432	4.4581439
Austria	1992	1.827177	4.4623625
Austria	1993	1.8145032	4.4610631
Austria	1994	1.8158412	4.4697005
Austria	1995	1.8061523	4.4804706
Austria	1996	1.8073905	4.4904668
Austria	1997	1.7999365	4.4998881

Austria	1998	1.8037919	4.5155484
Austria	1999	1.7995173	4.5298079
Austria	2000	1.7984149	4.5444066
Austria	2001	1.7964238	4.5464519
Austria	2002	1.7951264	4.5516094
Austria	2003	1.8003314	4.5532383
Austria	2004	1.7996193	4.5616471
Austria	2005	1.799086	4.5689912
Austria	2006	1.7967115	4.5824946
Austria	2007	1.7938917	4.5966131
Austria	2008	1.7943895	4.6009197
Bangladesh	1990	1.8037483	2.4307635
Bangladesh	1991	1.8000026	2.4348419
Bangladesh	1992	1.7963428	2.4464415
Bangladesh	1993	1.792373	2.4564419
Bangladesh	1994	1.7886349	2.4645844
Bangladesh	1995	1.7852784	2.4763098
Bangladesh	1996	1.7816005	2.4868571
Bangladesh	1997	1.7779831	2.500711
Bangladesh	1998	1.7742425	2.5141233
Bangladesh	1999	1.7707371	2.5263759
Bangladesh	2000	1.7677322	2.5434538
Bangladesh	2001	1.7651739	2.5581454
Bangladesh	2002	1.7619431	2.5696322
Bangladesh	2003	1.7599325	2.585024
Bangladesh	2004	1.7572924	2.6050898
Bangladesh	2005	1.7547186	2.6244093
Bangladesh	2006	1.7521587	2.6470578
Bangladesh	2007	1.7497738	2.6693773
Bangladesh	2008	1.7473916	2.6909994
Belgium	1990	1.8589992	4.4483412
Belgium	1991	1.8634792	4.4546163
Belgium	1992	1.8592181	4.4594515
Belgium	1993	1.849296	4.4535574
Belgium	1994	1.8515203	4.4660121
Belgium	1995	1.8549562	4.4753378
Belgium	1996	1.8524248	4.4806317
Belgium	1997	1.855362	4.4955067
Belgium	1998	1.8599861	4.502877
Belgium	1999	1.866174	4.5169898
Belgium	2000	1.8716951	4.531588
Belgium	2001	1.8684861	4.5335877
Belgium	2002	1.8731647	4.537506
Belgium	2003	1.856433	4.5391778
Belgium	2004	1.8713243	4.5512906
Belgium	2005	1.8602803	4.5564408
Belgium	2006	1.8714968	4.5650054

Belgium	2007	1.8734779	4.5741607
Belgium	2008	1.8760147	4.5749875
Bolivia	1990	1.8195954	2.9210957
Bolivia	1991	1.8159766	2.9331089
Bolivia	1992	1.8134772	2.929926
Bolivia	1993	1.8118041	2.9378723
Bolivia	1994	1.8089779	2.9476158
Bolivia	1995	1.8064326	2.9576011
Bolivia	1996	1.8027189	2.9664888
Bolivia	1997	1.8019033	2.9780479
Bolivia	1998	1.7998799	2.9901119
Bolivia	1999	1.7970286	2.9828819
Bolivia	2000	1.7923597	2.9847107
Bolivia	2001	1.7885099	2.9831674
Bolivia	2002	1.7860805	2.9851856
Bolivia	2003	1.7860329	2.988369
Bolivia	2004	1.781604	2.9979746
Bolivia	2005	1.7795372	3.0089365
Bolivia	2006	1.7781607	3.0217976
Bolivia	2007	1.7760312	3.0339832
Bolivia	2008	1.7736347	3.0528788
Botswana	1990	1.7984538	3.5717876
Botswana	1991	1.8019612	3.5904713
Botswana	1992	1.8161957	3.5907661
Botswana	1993	1.8248173	3.587244
Botswana	1994	1.8334554	3.5914541
Botswana	1995	1.8434609	3.6102467
Botswana	1996	1.851836	3.6246798
Botswana	1997	1.8638667	3.6485764
Botswana	1998	1.8794473	3.6426763
Botswana	1999	1.8887483	3.6744216
Botswana	2000	1.9246705	3.6753694
Botswana	2001	1.930433	3.6695899
Botswana	2002	1.9153695	3.6889841
Botswana	2003	1.9637981	3.7029879
Botswana	2004	1.9557261	3.7093683
Botswana	2005	1.952995	3.7238151
Botswana	2006	1.9512044	3.7524382
Botswana	2007	1.9534826	3.7842115
Botswana	2008	1.9581735	3.796651
Brazil	1990	1.7735999	3.601998
Brazil	1991	1.7712405	3.6013055
Brazil	1992	1.7680351	3.5923512
Brazil	1993	1.7665439	3.6054267
Brazil	1994	1.7650079	3.6213492
Brazil	1995	1.7651888	3.6334755
Brazil	1996	1.76443	3.6360543

Brazil	1997	1.7631238	3.6438288
Brazil	1998	1.7599202	3.6374067
Brazil	1999	1.7564935	3.6320697
Brazil	2000	1.7550126	3.6441148
Brazil	2001	1.752564	3.6437005
Brazil	2002	1.74944	3.6491726
Brazil	2003	1.7462967	3.6484504
Brazil	2004	1.745097	3.6672219
Brazil	2005	1.7441681	3.6757147
Brazil	2006	1.7432708	3.6879391
Brazil	2007	1.7430834	3.7093572
Brazil	2008	1.7427085	3.7272223
Cambodia	1990	1.8426108	2.3242825
Cambodia	1991	1.8385662	2.3443923
Cambodia	1992	1.8339687	2.363612
Cambodia	1993	1.8292688	2.3834241
Cambodia	1994	1.824668	2.4063
Cambodia	1995	1.8203973	2.4195288
Cambodia	1996	1.8154636	2.4296481
Cambodia	1997	1.8097539	2.4415981
Cambodia	1998	1.8042507	2.4519345
Cambodia	1999	1.7977085	2.4906954
Cambodia	2000	1.7911324	2.5177666
Cambodia	2001	1.7846497	2.5425624
Cambodia	2002	1.7768553	2.5625111
Cambodia	2003	1.772092	2.5903421
Cambodia	2004	1.7655338	2.6259169
Cambodia	2005	1.7599192	2.6731707
Cambodia	2006	1.7543478	2.7111861
Cambodia	2007	1.7492864	2.7473014
Cambodia	2008	1.7449578	2.7693697
Canada	1990	1.8467938	4.4310909
Canada	1991	1.8459355	4.4159997
Canada	1992	1.8476904	4.4144535
Canada	1993	1.8456792	4.4197401
Canada	1994	1.8374536	4.4359442
Canada	1995	1.8326818	4.4443762
Canada	1996	1.830083	4.4466722
Canada	1997	1.8365027	4.4600574
Canada	1998	1.8325155	4.4737381
Canada	1999	1.8302544	4.4935281
Canada	2000	1.8378159	4.5118464
Canada	2001	1.8320216	4.5151408
Canada	2002	1.832222	4.5237635
Canada	2003	1.8311693	4.5275304
Canada	2004	1.8341497	4.5365214
Canada	2005	1.8359241	4.5451573

Canada	2006	1.8348639	4.553787
Canada	2007	1.8421674	4.559026
Canada	2008	1.8426417	4.5573069
Chile	1990	1.7408659	3.6150383
Chile	1991	1.7378726	3.640442
Chile	1992	1.7372932	3.6827416
Chile	1993	1.7369224	3.7041609
Chile	1994	1.7367184	3.7206713
Chile	1995	1.737786	3.7573973
Chile	1996	1.7403081	3.7818236
Chile	1997	1.7424555	3.8034236
Chile	1998	1.7394547	3.8114333
Chile	1999	1.7391441	3.8025869
Chile	2000	1.7364089	3.8163456
Chile	2001	1.7277594	3.8254836
Chile	2002	1.7287255	3.8298175
Chile	2003	1.7243609	3.8418615
Chile	2004	1.724874	3.862675
Chile	2005	1.7259457	3.8816426
Chile	2006	1.7258876	3.8959529
Chile	2007	1.7265634	3.9135033
Chile	2008	1.7262487	3.923364
China	1990	1.7592878	2.6656569
China	1991	1.7585752	2.6979539
China	1992	1.7580397	2.7502975
China	1993	1.7582412	2.8022097
China	1994	1.7572503	2.8507636
China	1995	1.7576053	2.8909765
China	1996	1.7576673	2.9278172
China	1997	1.7547327	2.9619925
China	1998	1.7511868	2.990444
China	1999	1.750345	3.0184959
China	2000	1.7475979	3.0501032
China	2001	1.7457599	3.081577
China	2002	1.7438815	3.116492
China	2003	1.7455313	3.1551796
China	2004	1.7467217	3.1943875
China	2005	1.7486309	3.2383285
China	2006	1.7495845	3.2878274
China	2007	1.7511677	3.3432253
China	2008	1.7539082	3.3808106
Colombia	1990	1.7655191	3.4521647
Colombia	1991	1.763633	3.4535454
Colombia	1992	1.7637207	3.4666242
Colombia	1993	1.7636404	3.4686691
Colombia	1994	1.7632548	3.4853086
Colombia	1995	1.7590503	3.4994443

Colombia	1996	1.7567627	3.500489
Colombia	1997	1.7557336	3.5074464
Colombia	1998	1.7533662	3.502341
Colombia	1999	1.7464644	3.4762522
Colombia	2000	1.7445654	3.4877487
Colombia	2001	1.7424927	3.4878428
Colombia	2002	1.7411601	3.4915979
Colombia	2003	1.7397208	3.5014347
Colombia	2004	1.737397	3.5172523
Colombia	2005	1.7375435	3.5305742
Colombia	2006	1.7373955	3.5521675
Colombia	2007	1.7364626	3.5746876
Colombia	2008	1.7349435	3.5834816
Costa Rica	1990	1.7171963	3.5035427
Costa Rica	1991	1.7161818	3.5037834
Costa Rica	1992	1.7155165	3.5312673
Costa Rica	1993	1.7152722	3.5518783
Costa Rica	1994	1.7195808	3.5614209
Costa Rica	1995	1.7162542	3.567427
Costa Rica	1996	1.7143167	3.5603596
Costa Rica	1997	1.7151774	3.5729427
Costa Rica	1998	1.7154122	3.5971104
Costa Rica	1999	1.7167655	3.6209985
Costa Rica	2000	1.7144078	3.6189301
Costa Rica	2001	1.7142959	3.6144049
Costa Rica	2002	1.7182449	3.6182206
Costa Rica	2003	1.7149723	3.6370562
Costa Rica	2004	1.7155999	3.6474126
Costa Rica	2005	1.7139863	3.6647702
Costa Rica	2006	1.7133667	3.6941035
Costa Rica	2007	1.7128765	3.7203294
Costa Rica	2008	1.7122502	3.7253244
Denmark	1990	1.8187866	4.561624
Denmark	1991	1.8249841	4.5661082
Denmark	1992	1.8174719	4.5731678
Denmark	1993	1.8207257	4.5713315
Denmark	1994	1.8198878	4.5932219
Denmark	1995	1.8174757	4.6040713
Denmark	1996	1.8226637	4.6137525
Denmark	1997	1.8104947	4.625621
Denmark	1998	1.8089567	4.6333266
Denmark	1999	1.805072	4.6428695
Denmark	2000	1.796814	4.6564785
Denmark	2001	1.7951928	4.6579727
Denmark	2002	1.796764	4.6586036
Denmark	2003	1.8008441	4.6590861
Denmark	2004	1.8041923	4.6678244

Denmark	2005	1.797989	4.6771194
Denmark	2006	1.8018511	4.6901905
Denmark	2007	1.7987753	4.6950867
Denmark	2008	1.7943486	4.6891173
Ecuador	1990	1.759061	3.4228692
Ecuador	1991	1.7553765	3.431066
Ecuador	1992	1.7561016	3.4303079
Ecuador	1993	1.7557226	3.4291428
Ecuador	1994	1.7451428	3.4377738
Ecuador	1995	1.7489197	3.4381242
Ecuador	1996	1.746298	3.4364152
Ecuador	1997	1.7398058	3.4458104
Ecuador	1998	1.7412558	3.4509108
Ecuador	1999	1.7350511	3.4210935
Ecuador	2000	1.7317608	3.4171971
Ecuador	2001	1.734111	3.4257934
Ecuador	2002	1.7340468	3.4348453
Ecuador	2003	1.7315234	3.4382665
Ecuador	2004	1.7337492	3.4644611
Ecuador	2005	1.7332798	3.4789634
Ecuador	2006	1.7329166	3.4899809
Ecuador	2007	1.7326596	3.4918709
Ecuador	2008	1.7317583	3.5112734
Egypt	1990	1.7834999	2.9440687
Egypt	1991	1.7807732	2.9407017
Egypt	1992	1.7783782	2.9521583
Egypt	1993	1.7773758	2.9576603
Egypt	1994	1.7744909	2.9678669
Egypt	1995	1.7732161	2.9808741
Egypt	1996	1.7709941	2.9952873
Egypt	1997	1.768787	3.0117713
Egypt	1998	1.7690028	3.0221924
Egypt	1999	1.7679687	3.0411166
Egypt	2000	1.7671538	3.0569497
Egypt	2001	1.7628937	3.0650833
Egypt	2002	1.7635303	3.0682152
Egypt	2003	1.7619027	3.0747442
Egypt	2004	1.7604507	3.0849734
Egypt	2005	1.7630029	3.0967339
Egypt	2006	1.76046	3.118205
Egypt	2007	1.7601261	3.1406359
Egypt	2008	1.7617171	3.1633301
Ethiopia	1990	1.908912	2.1503562
Ethiopia	1991	1.9051844	2.1030857
Ethiopia	1992	1.9013543	2.0484021
Ethiopia	1993	1.8978439	2.0868543
Ethiopia	1994	1.8928601	2.0857525

Ethiopia	1995	1.8879645	2.0974651
Ethiopia	1996	1.8832658	2.1348966
Ethiopia	1997	1.8783358	2.1354278
Ethiopia	1998	1.8734742	2.1076392
Ethiopia	1999	1.8682831	2.1171068
Ethiopia	2000	1.8629113	2.1302648
Ethiopia	2001	1.8568651	2.1523688
Ethiopia	2002	1.8504598	2.1463537
Ethiopia	2003	1.8436053	2.1243726
Ethiopia	2004	1.8362776	2.1672957
Ethiopia	2005	1.8285536	2.2036622
Ethiopia	2006	1.8207527	2.236396
Ethiopia	2007	1.8130148	2.2717335
Ethiopia	2008	1.8056864	2.3046129
Finland	1990	1.8824204	4.4490596
Finland	1991	1.8857889	4.4198158
Finland	1992	1.8730451	4.4019703
Finland	1993	1.8478563	4.3963338
Finland	1994	1.8454642	4.4100467
Finland	1995	1.8316337	4.4252657
Finland	1996	1.8307743	4.4390727
Finland	1997	1.8270928	4.4639294
Finland	1998	1.8237263	4.4840942
Finland	1999	1.819736	4.4997367
Finland	2000	1.8198208	4.5213613
Finland	2001	1.8204897	4.5301791
Finland	2002	1.8204025	4.5370199
Finland	2003	1.8346973	4.5446374
Finland	2004	1.8396096	4.5609309
Finland	2005	1.8239887	4.5719276
Finland	2006	1.8357614	4.5890055
Finland	2007	1.8302476	4.6097315
Finland	2008	1.8246704	4.6089827
France	1990	1.7939949	4.4440612
France	1991	1.7965174	4.4474537
France	1992	1.7893755	4.4516641
France	1993	1.7808488	4.4468721
France	1994	1.7763357	4.4549055
France	1995	1.7783119	4.4621365
France	1996	1.7768103	4.46521
France	1997	1.7695963	4.4730557
France	1998	1.7725587	4.4858845
France	1999	1.7666016	4.4977177
France	2000	1.7661078	4.5104399
France	2001	1.7659479	4.5151809
France	2002	1.7649909	4.5160401
France	2003	1.7661305	4.5168534

France	2004	1.7646439	4.5245712
France	2005	1.7636459	4.5291604
France	2006	1.7578054	4.5367178
France	2007	1.7559585	4.5438452
France	2008	1.7561467	4.5410691
Germany	1990	1.8542495	4.4456177
Germany	1991	1.8482246	4.4640903
Germany	1992	1.8434892	4.469013
Germany	1993	1.838136	4.461784
Germany	1994	1.8338018	4.4708817
Germany	1995	1.8317023	4.4768281
Germany	1996	1.8269665	4.4789917
Germany	1997	1.8201434	4.4858364
Germany	1998	1.8180002	4.4937822
Germany	1999	1.8074166	4.5015524
Germany	2000	1.8066745	4.5140444
Germany	2001	1.8037136	4.5198414
Germany	2002	1.801152	4.5191553
Germany	2003	1.8053755	4.5172812
Germany	2004	1.8059316	4.5223892
Germany	2005	1.8011738	4.5255991
Germany	2006	1.8022149	4.5418677
Germany	2007	1.798439	4.5564186
Germany	2008	1.7981937	4.5619234
Greece	1990	1.7805653	4.1870703
Greece	1991	1.7750803	4.1960999
Greece	1992	1.7770414	4.1943466
Greece	1993	1.775294	4.1833537
Greece	1994	1.7748461	4.1883376
Greece	1995	1.7768199	4.1940272
Greece	1996	1.7758053	4.2011071
Greece	1997	1.7719037	4.2139025
Greece	1998	1.7770335	4.2259243
Greece	1999	1.7793062	4.2386185
Greece	2000	1.786888	4.2562503
Greece	2001	1.7852957	4.2728163
Greece	2002	1.784342	4.2860142
Greece	2003	1.786968	4.3096702
Greece	2004	1.7874095	4.326734
Greece	2005	1.7871636	4.3348701
Greece	2006	1.7865369	4.3564167
Greece	2007	1.788524	4.3697864
Greece	2008	1.7864221	4.3671345
Guatemala	1990	1.7938064	3.2459079
Guatemala	1991	1.7899558	3.2514544
Guatemala	1992	1.7870017	3.2618641
Guatemala	1993	1.7840685	3.2684696

Guatemala	1994	1.7812836	3.2755634
Guatemala	1995	1.7775195	3.2865308
Guatemala	1996	1.7731598	3.2892661
Guatemala	1997	1.7707754	3.2979384
Guatemala	1998	1.7685324	3.3091801
Guatemala	1999	1.7648554	3.315503
Guatemala	2000	1.7621773	3.320613
Guatemala	2001	1.7598797	3.3201036
Guatemala	2002	1.7586699	3.3258639
Guatemala	2003	1.7539825	3.3258882
Guatemala	2004	1.7523888	3.3285229
Guatemala	2005	1.750933	3.331666
Guatemala	2006	1.7491346	3.3437165
Guatemala	2007	1.7476129	3.3596184
Guatemala	2008	1.7458215	3.3629972
Hong Kong	1990	1.7084975	4.2446753
Hong Kong	1991	1.7058833	4.265157
Hong Kong	1992	1.708766	4.2877778
Hong Kong	1993	1.892822	4.3064472
Hong Kong	1994	1.9059931	4.3221203
Hong Kong	1995	1.8971975	4.3237093
Hong Kong	1996	1.866705	4.3225443
Hong Kong	1997	1.8740005	4.3405297
Hong Kong	1998	1.842131	4.3105743
Hong Kong	1999	1.8249637	4.3171783
Hong Kong	2000	1.8272064	4.3454178
Hong Kong	2001	1.8339212	4.3446463
Hong Kong	2002	1.8151705	4.3498591
Hong Kong	2003	1.7979987	4.363791
Hong Kong	2004	1.7920569	4.3966338
Hong Kong	2005	1.7963991	4.4256932
Hong Kong	2006	1.7884947	4.45242
Hong Kong	2007	1.7955643	4.4758927
Hong Kong	2008	1.7955974	4.4824389
India	1990	1.821134	2.6054023
India	1991	1.8191795	2.6013286
India	1992	1.8171875	2.616065
India	1993	1.8148488	2.6279601
India	1994	1.8128311	2.6478553
India	1995	1.8105283	2.6716079
India	1996	1.8082616	2.6954067
India	1997	1.8057026	2.7049897
India	1998	1.8029744	2.7235362
India	1999	1.8009029	2.7529689
India	2000	1.7978516	2.762091
India	2001	1.795193	2.7754271
India	2002	1.792556	2.7846454

India	2003	1.7902802	2.8106717
India	2004	1.7880191	2.83715
India	2005	1.785692	2.8692988
India	2006	1.7838211	2.9015988
India	2007	1.7825359	2.9362436
India	2008	1.7811469	2.9470274
Indonesia	1990	1.7861019	2.9243933
Indonesia	1991	1.7844799	2.9539331
Indonesia	1992	1.7823193	2.9767956
Indonesia	1993	1.7804846	2.9999835
Indonesia	1994	1.7782214	3.0245281
Indonesia	1995	1.7761442	3.0527173
Indonesia	1996	1.7743055	3.0780739
Indonesia	1997	1.7725372	3.0915625
Indonesia	1998	1.7639295	3.0241116
Indonesia	1999	1.7652034	3.0212554
Indonesia	2000	1.7640102	3.0358502
Indonesia	2001	1.7625765	3.0451358
Indonesia	2002	1.7618329	3.0580093
Indonesia	2003	1.7599164	3.0720585
Indonesia	2004	1.7592989	3.0871528
Indonesia	2005	1.7578533	3.1049871
Indonesia	2006	1.7560683	3.1220411
Indonesia	2007	1.7549931	3.1425792
Indonesia	2008	1.7538019	3.1618345
Iran, Islamic Republic of	1990	1.8206382	3.2555769
Iran, Islamic Republic of	1991	1.8125637	3.2986204
Iran, Islamic Republic of	1992	1.807398	3.3104354
Iran, Islamic Republic of	1993	1.7944484	3.2985387
Iran, Islamic Republic of	1994	1.7985244	3.2920304
Iran, Islamic Republic of	1995	1.7950864	3.2975785
Iran, Islamic Republic of	1996	1.7932739	3.3204416
Iran, Islamic Republic of	1997	1.7925648	3.3271803
Iran, Islamic Republic of	1998	1.7943582	3.3308671
Iran, Islamic Republic of	1999	1.7851326	3.3314475
Iran, Islamic Republic of	2000	1.7847488	3.3462389
Iran, Islamic Republic of	2001	1.7848109	3.3556979
Iran, Islamic Republic of	2002	1.7803781	3.3815568
Iran, Islamic Republic of	2003	1.7900831	3.4062052
Iran, Islamic Republic of	2004	1.7904486	3.422709
Iran, Islamic Republic of	2005	1.7890494	3.4372926
Iran, Islamic Republic of	2006	1.7880901	3.457091
Iran, Islamic Republic of	2007	1.7867478	3.4847445
Iran, Islamic Republic of	2008	1.7872346	3.4821357
Ireland	1990	1.8224694	4.3460686
Ireland	1991	1.8259363	4.3518722
Ireland	1992	1.8194632	4.3631914

Ireland	1993	1.8143491	4.3725598
Ireland	1994	1.8161464	4.3951522
Ireland	1995	1.8197297	4.4328725
Ireland	1996	1.8368645	4.4696506
Ireland	1997	1.8414463	4.5116803
Ireland	1998	1.8448385	4.5443024
Ireland	1999	1.8412909	4.5846173
Ireland	2000	1.8377638	4.6227729
Ireland	2001	1.8344159	4.6369857
Ireland	2002	1.8309473	4.6525785
Ireland	2003	1.8442531	4.6614073
Ireland	2004	1.8462986	4.6713339
Ireland	2005	1.844122	4.6875074
Ireland	2006	1.8385439	4.6990171
Ireland	2007	1.8337507	4.7075832
Ireland	2008	1.8285739	4.689244
Italy	1990	1.8011076	4.4078352
Italy	1991	1.8034718	4.4141651
Italy	1992	1.8001483	4.4174783
Italy	1993	1.7857324	4.4134932
Italy	1994	1.7825944	4.4226474
Italy	1995	1.7860935	4.4350003
Italy	1996	1.776955	4.4397784
Italy	1997	1.775424	4.4475779
Italy	1998	1.7752051	4.4536972
Italy	1999	1.7727065	4.4598809
Italy	2000	1.7755629	4.4752685
Italy	2001	1.7719994	4.4830157
Italy	2002	1.7732765	4.4836005
Italy	2003	1.7755155	4.4800131
Italy	2004	1.774075	4.4831839
Italy	2005	1.7761008	4.4839985
Italy	2006	1.7753435	4.4909735
Italy	2007	1.7733086	4.4950375
Italy	2008	1.7708234	4.4866577
Japan	1990	1.7950736	4.4938067
Japan	1991	1.7955403	4.5066615
Japan	1992	1.79731	4.5091259
Japan	1993	1.7947622	4.5087963
Japan	1994	1.7971803	4.5110511
Japan	1995	1.8006012	4.5177476
Japan	1996	1.7956022	4.5278241
Japan	1997	1.7932132	4.5335611
Japan	1998	1.7859524	4.5236758
Japan	1999	1.7848139	4.5219855
Japan	2000	1.7880675	4.5309269
Japan	2001	1.7833829	4.5315139

Japan	2002	1.7823536	4.5317597
Japan	2003	1.7824557	4.5380878
Japan	2004	1.7839209	4.548075
Japan	2005	1.783206	4.5536553
Japan	2006	1.7794488	4.5610038
Japan	2007	1.7796993	4.5703713
Japan	2008	1.7809374	4.5660506
Korea	1990	1.7984443	3.9429224
Korea	1991	1.8012006	3.9776141
Korea	1992	1.8022423	3.9978973
Korea	1993	1.8060747	4.0193393
Korea	1994	1.810549	4.0505455
Korea	1995	1.8163774	4.0842748
Korea	1996	1.8199228	4.1095151
Korea	1997	1.8169168	4.1251858
Korea	1998	1.7857261	4.0912129
Korea	1999	1.7974519	4.1274858
Korea	2000	1.8064204	4.1592288
Korea	2001	1.7998019	4.1729367
Korea	2002	1.8064462	4.2005072
Korea	2003	1.8025044	4.2103548
Korea	2004	1.8030159	4.2283342
Korea	2005	1.802279	4.2442976
Korea	2006	1.8026488	4.2641205
Korea	2007	1.8056059	4.2837255
Korea	2008	1.8006638	4.2904684
Lao Peoples Democratic Republic	1990	1.8479367	2.4175368
Lao Peoples Democratic Republic	1991	1.8418333	2.4231599
Lao Peoples Democratic Republic	1992	1.8356346	2.4341314
Lao Peoples Democratic Republic	1993	1.8294953	2.4468891
Lao Peoples Democratic Republic	1994	1.823852	2.4693464
Lao Peoples Democratic Republic	1995	1.8181102	2.4879948
Lao Peoples Democratic Republic	1996	1.8127391	2.5069439
Lao Peoples Democratic Republic	1997	1.8075022	2.5263209
Lao Peoples Democratic Republic	1998	1.8025941	2.5344318
Lao Peoples Democratic Republic	1999	1.7973375	2.5570117
Lao Peoples Democratic Republic	2000	1.7941973	2.5741661
Lao Peoples Democratic Republic	2001	1.7891883	2.5919017
Lao Peoples Democratic Republic	2002	1.7857324	2.6109546
Lao Peoples Democratic Republic	2003	1.7815143	2.6307912
Lao Peoples Democratic Republic	2004	1.7780916	2.6514173
Lao Peoples Democratic Republic	2005	1.7746576	2.6743177
Lao Peoples Democratic Republic	2006	1.771576	2.7023992
Lao Peoples Democratic Republic	2007	1.7685062	2.7256397
Lao Peoples Democratic Republic	2008	1.7654234	2.7493635
Madagascar	1990	1.8734408	2.5164255
Madagascar	1991	1.8684822	2.4750605

Madagascar	1992	1.8627357	2.4670368
Madagascar	1993	1.8567428	2.4627966
Madagascar	1994	1.8505085	2.4490959
Madagascar	1995	1.8440082	2.4429346
Madagascar	1996	1.837323	2.4384999
Madagascar	1997	1.8313374	2.4405006
Madagascar	1998	1.8254759	2.443499
Madagascar	1999	1.8200584	2.4496168
Madagascar	2000	1.814698	2.4563098
Madagascar	2001	1.8100603	2.4683533
Madagascar	2002	1.8056376	2.3963183
Madagascar	2003	1.8023734	2.4238432
Madagascar	2004	1.7984317	2.433239
Madagascar	2005	1.7956253	2.4400849
Madagascar	2006	1.7925024	2.4488297
Madagascar	2007	1.7895502	2.4627291
Madagascar	2008	1.7866392	2.4803488
Malawi	1990	1.9084696	2.2767282
Malawi	1991	1.9079482	2.3035126
Malawi	1992	1.908162	2.2658787
Malawi	1993	1.9091327	2.3042234
Malawi	1994	1.910772	2.2550468
Malawi	1995	1.9125087	2.3173011
Malawi	1996	1.9148208	2.3397971
Malawi	1997	1.9169264	2.3453594
Malawi	1998	1.918299	2.3497752
Malawi	1999	1.9195223	2.3502876
Malawi	2000	1.9185074	2.3450693
Malawi	2001	1.9161542	2.3114868
Malawi	2002	1.9121101	2.307609
Malawi	2003	1.9064942	2.3196479
Malawi	2004	1.8996923	2.328656
Malawi	2005	1.8918672	2.3286989
Malawi	2006	1.8834949	2.3248874
Malawi	2007	1.8750713	2.3512086
Malawi	2008	1.8671781	2.3727553
Malaysia	1990	1.7684106	3.4979089
Malaysia	1991	1.7706978	3.5257665
Malaysia	1992	1.7710097	3.5513911
Malaysia	1993	1.7749237	3.5812883
Malaysia	1994	1.774498	3.6085641
Malaysia	1995	1.7816108	3.6382712
Malaysia	1996	1.78112	3.6686171
Malaysia	1997	1.7822321	3.6882959
Malaysia	1998	1.7628108	3.6442942
Malaysia	1999	1.7633573	3.6597787
Malaysia	2000	1.7680941	3.6868022

Malaysia	2001	1.7687439	3.6797801
Malaysia	2002	1.770643	3.6938137
Malaysia	2003	1.7735332	3.7098511
Malaysia	2004	1.7750997	3.7301549
Malaysia	2005	1.7754908	3.7446015
Malaysia	2006	1.7769756	3.7601516
Malaysia	2007	1.7783097	3.7787227
Malaysia	2008	1.7770067	3.7913757
Mauritius	1990	1.7699071	3.4825107
Mauritius	1991	1.7669214	3.4966706
Mauritius	1992	1.7673884	3.5183578
Mauritius	1993	1.7684593	3.5347376
Mauritius	1994	1.7661151	3.5462587
Mauritius	1995	1.764729	3.5607573
Mauritius	1996	1.7683344	3.5799294
Mauritius	1997	1.7676842	3.5985152
Mauritius	1998	1.7661413	3.619544
Mauritius	1999	1.7675207	3.6252161
Mauritius	2000	1.767819	3.6584809
Mauritius	2001	1.7658445	3.6647824
Mauritius	2002	1.7627737	3.6701473
Mauritius	2003	1.7682482	3.6812417
Mauritius	2004	1.7689091	3.701765
Mauritius	2005	1.7676672	3.7036626
Mauritius	2006	1.7711458	3.7171904
Mauritius	2007	1.7714674	3.7393801
Mauritius	2008	1.7747186	3.7598787
Mexico	1990	1.7779865	3.8165722
Mexico	1991	1.774879	3.8254799
Mexico	1992	1.7744024	3.8318269
Mexico	1993	1.7685895	3.8400389
Mexico	1994	1.7678727	3.8512449
Mexico	1995	1.757793	3.8169761
Mexico	1996	1.7562947	3.8336103
Mexico	1997	1.756912	3.8550193
Mexico	1998	1.755528	3.8675325
Mexico	1999	1.7526759	3.8719549
Mexico	2000	1.7527534	3.8878104
Mexico	2001	1.7513211	3.8790852
Mexico	2002	1.7488098	3.8739898
Mexico	2003	1.7495658	3.8747354
Mexico	2004	1.7477622	3.887708
Mexico	2005	1.749247	3.8953541
Mexico	2006	1.7484731	3.9111513
Mexico	2007	1.7482643	3.9191734
Mexico	2008	1.7473305	3.9197455
Morocco	1990	1.7803776	3.1717341

Morocco	1991	1.7772565	3.1927504
Morocco	1992	1.7742484	3.1672615
Morocco	1993	1.7727548	3.1555321
Morocco	1994	1.7708014	3.1913027
Morocco	1995	1.76978	3.154968
Morocco	1996	1.7665113	3.198477
Morocco	1997	1.764292	3.1823767
Morocco	1998	1.7632615	3.2083821
Morocco	1999	1.7627417	3.204992
Morocco	2000	1.7616528	3.2065532
Morocco	2001	1.7612971	3.2332339
Morocco	2002	1.7610047	3.2427652
Morocco	2003	1.7590047	3.2649644
Morocco	2004	1.7587951	3.2810773
Morocco	2005	1.7601276	3.289634
Morocco	2006	1.7580806	3.3179855
Morocco	2007	1.756894	3.3255174
Morocco	2008	1.7557267	3.3449051
Mozambique	1990	1.9421348	2.2715673
Mozambique	1991	1.9386874	2.2820442
Mozambique	1992	1.9345209	2.2452362
Mozambique	1993	1.9301094	2.2651619
Mozambique	1994	1.9250474	2.2775555
Mozambique	1995	1.9208782	2.2745356
Mozambique	1996	1.9157739	2.2926391
Mozambique	1997	1.9117263	2.3232304
Mozambique	1998	1.9089209	2.3566447
Mozambique	1999	1.9077965	2.379486
Mozambique	2000	1.9064351	2.3727001
Mozambique	2001	1.9054599	2.4095702
Mozambique	2002	1.904783	2.4340899
Mozambique	2003	1.9040977	2.4472242
Mozambique	2004	1.9032552	2.4715516
Mozambique	2005	1.9026588	2.4956941
Mozambique	2006	1.9010792	2.5105492
Mozambique	2007	1.8991645	2.5294815
Mozambique	2008	1.8969705	2.5467459
Netherlands	1990	1.8270945	4.4666169
Netherlands	1991	1.828017	4.4736601
Netherlands	1992	1.8248193	4.4777234
Netherlands	1993	1.8283212	4.4801241
Netherlands	1994	1.8222766	4.4901787
Netherlands	1995	1.8285477	4.5013596
Netherlands	1996	1.8261393	4.5139037
Netherlands	1997	1.820302	4.5298626
Netherlands	1998	1.8204856	4.5438982
Netherlands	1999	1.8077894	4.5608899

Netherlands	2000	1.8060749	4.5745727
Netherlands	2001	1.8017145	4.5795789
Netherlands	2002	1.8095684	4.5771381
Netherlands	2003	1.8078652	4.5765441
Netherlands	2004	1.8069483	4.5846411
Netherlands	2005	1.8045495	4.5924243
Netherlands	2006	1.805869	4.6062229
Netherlands	2007	1.8033157	4.62198
Netherlands	2008	1.8039408	4.6280544
New Zealand	1990	1.7807393	4.3202415
New Zealand	1991	1.7740413	4.2943012
New Zealand	1992	1.7752591	4.2937654
New Zealand	1993	1.7731538	4.3149251
New Zealand	1994	1.774865	4.3299841
New Zealand	1995	1.7769105	4.3415105
New Zealand	1996	1.7818886	4.348936
New Zealand	1997	1.7807615	4.3558339
New Zealand	1998	1.769679	4.3580057
New Zealand	1999	1.7820976	4.3778102
New Zealand	2000	1.7753744	4.38557
New Zealand	2001	1.7747666	4.3989141
New Zealand	2002	1.7758143	4.4125484
New Zealand	2003	1.7721008	4.4212601
New Zealand	2004	1.7729777	4.4304265
New Zealand	2005	1.7721922	4.4399136
New Zealand	2006	1.7666582	4.4417279
New Zealand	2007	1.7659262	4.4522502
New Zealand	2008	1.7657931	4.4403835
Nicaragua	1990	1.7797748	2.9719249
Nicaragua	1991	1.7733937	2.9607899
Nicaragua	1992	1.7696533	2.9518133
Nicaragua	1993	1.7655626	2.9394367
Nicaragua	1994	1.7625328	2.9434004
Nicaragua	1995	1.7595249	2.9587008
Nicaragua	1996	1.7566733	2.9764508
Nicaragua	1997	1.7541478	2.9849678
Nicaragua	1998	1.7521206	2.9929807
Nicaragua	1999	1.7498455	3.0152047
Nicaragua	2000	1.7476264	3.0258106
Nicaragua	2001	1.744941	3.0320795
Nicaragua	2002	1.7419813	3.0293517
Nicaragua	2003	1.7399034	3.0344666
Nicaragua	2004	1.7371419	3.051393
Nicaragua	2005	1.73435	3.0640823
Nicaragua	2006	1.7320723	3.0762359
Nicaragua	2007	1.7297731	3.0920301
Nicaragua	2008	1.7274229	3.1034571

Nigeria	1990	1.9209319	2.7708902
Nigeria	1991	1.9208685	2.7571311
Nigeria	1992	1.9227167	2.7480504
Nigeria	1993	1.9222484	2.7461526
Nigeria	1994	1.9210402	2.739241
Nigeria	1995	1.9196138	2.7270668
Nigeria	1996	1.9195761	2.737386
Nigeria	1997	1.9191776	2.7385348
Nigeria	1998	1.9182751	2.7393047
Nigeria	1999	1.915468	2.7304803
Nigeria	2000	1.9142248	2.7420861
Nigeria	2001	1.9130816	2.7499145
Nigeria	2002	1.9110335	2.75509
Nigeria	2003	1.9076211	2.786844
Nigeria	2004	1.9040284	2.9019352
Nigeria	2005	1.9000362	2.9053383
Nigeria	2006	1.8928674	2.9281598
Nigeria	2007	1.8887227	2.9452674
Nigeria	2008	1.8834409	2.9599746
Norway	1990	1.850308	4.6502068
Norway	1991	1.847765	4.661419
Norway	1992	1.8392806	4.6739498
Norway	1993	1.8334117	4.6833012
Norway	1994	1.8320339	4.7022303
Norway	1995	1.8274307	4.7177866
Norway	1996	1.823535	4.737187
Norway	1997	1.8233023	4.7576424
Norway	1998	1.8274833	4.766554
Norway	1999	1.8278529	4.7722901
Norway	2000	1.820848	4.7833765
Norway	2001	1.8232984	4.7897365
Norway	2002	1.8182894	4.7938694
Norway	2003	1.824786	4.7955613
Norway	2004	1.8342154	4.8098655
Norway	2005	1.8560006	4.8180082
Norway	2006	1.8210925	4.8243808
Norway	2007	1.8207179	4.8312588
Norway	2008	1.8176356	4.8261394
Pakistan	1990	1.8008774	2.7200968
Pakistan	1991	1.7986035	2.7294421
Pakistan	1992	1.7969098	2.7501926
Pakistan	1993	1.795058	2.7466279
Pakistan	1994	1.7936229	2.7514671
Pakistan	1995	1.7916757	2.7612552
Pakistan	1996	1.7905736	2.7703275
Pakistan	1997	1.7883969	2.7631354
Pakistan	1998	1.7863026	2.7627084

Pakistan	1999	1.7848456	2.7675573
Pakistan	2000	1.7833079	2.7757446
Pakistan	2001	1.7811596	2.7752309
Pakistan	2002	1.7796053	2.7807048
Pakistan	2003	1.7780059	2.7934196
Pakistan	2004	1.7776444	2.8165829
Pakistan	2005	1.7766715	2.8408439
Pakistan	2006	1.7758835	2.8588834
Pakistan	2007	1.7753386	2.8712984
Pakistan	2008	1.7745282	2.8705418
Panama	1990	1.7288847	3.487633
Panama	1991	1.7273762	3.5176758
Panama	1992	1.7269471	3.5429419
Panama	1993	1.7260395	3.5570897
Panama	1994	1.726101	3.5603755
Panama	1995	1.7236465	3.5589758
Panama	1996	1.7252265	3.5620459
Panama	1997	1.7257021	3.5802624
Panama	1998	1.7260749	3.6020823
Panama	1999	1.7273243	3.6098906
Panama	2000	1.725503	3.6127476
Panama	2001	1.7247478	3.6065646
Panama	2002	1.7233223	3.6075773
Panama	2003	1.7162959	3.6170315
Panama	2004	1.7175954	3.6402294
Panama	2005	1.7116539	3.6622365
Panama	2006	1.71153	3.689777
Panama	2007	1.7104251	3.7315829
Panama	2008	1.7129181	3.7657391
Paraguay	1990	1.7550078	3.1685094
Paraguay	1991	1.7536919	3.1722641
Paraguay	1992	1.7532441	3.1686937
Paraguay	1993	1.7531448	3.1790207
Paraguay	1994	1.7541858	3.1911904
Paraguay	1995	1.7537381	3.209777
Paraguay	1996	1.7513801	3.2067339
Paraguay	1997	1.7512611	3.215197
Paraguay	1998	1.749163	3.2061275
Paraguay	1999	1.7475745	3.1909745
Paraguay	2000	1.744855	3.1717898
Paraguay	2001	1.743241	3.1592846
Paraguay	2002	1.7401535	3.1504716
Paraguay	2003	1.7394259	3.1602764
Paraguay	2004	1.7382803	3.1691491
Paraguay	2005	1.7366208	3.1700867
Paraguay	2006	1.7361799	3.1824165
Paraguay	2007	1.7354097	3.1974397

Paraguay	2008	1.7345649	3.2164343
Peru	1990	1.7748791	3.3034333
Peru	1991	1.7712699	3.304024
Peru	1992	1.7677911	3.2937114
Peru	1993	1.7661284	3.3057199
Peru	1994	1.7628724	3.350103
Peru	1995	1.7601831	3.3781355
Peru	1996	1.7568002	3.3812633
Peru	1997	1.7547009	3.4026049
Peru	1998	1.7517358	3.392494
Peru	1999	1.7492275	3.3895293
Peru	2000	1.7465276	3.3956182
Peru	2001	1.7423133	3.3903762
Peru	2002	1.7403203	3.4058004
Peru	2003	1.7366551	3.4174243
Peru	2004	1.7361196	3.4332476
Peru	2005	1.7354286	3.4568942
Peru	2006	1.7340471	3.484484
Peru	2007	1.7335462	3.516798
Peru	2008	1.7333579	3.5527175
Philippines	1990	1.7755446	3.0010567
Philippines	1991	1.7742588	2.9879572
Philippines	1992	1.7736958	2.9791436
Philippines	1993	1.7729298	2.9781955
Philippines	1994	1.7728655	2.9869444
Philippines	1995	1.773404	2.9969859
Philippines	1996	1.7722062	3.0119231
Philippines	1997	1.7723337	3.0242476
Philippines	1998	1.7696454	3.0122204
Philippines	1999	1.7686795	3.0160193
Philippines	2000	1.7687505	3.0255312
Philippines	2001	1.7671882	3.0288121
Philippines	2002	1.7660342	3.0353886
Philippines	2003	1.7645158	3.0476871
Philippines	2004	1.763678	3.0673729
Philippines	2005	1.7628358	3.0795207
Philippines	2006	1.7609211	3.093963
Philippines	2007	1.7605616	3.1143511
Philippines	2008	1.7595111	3.1247737
Portugal	1990	1.7714964	4.1388527
Portugal	1991	1.7752171	4.1584219
Portugal	1992	1.777613	4.1634655
Portugal	1993	1.7697447	4.1539685
Portugal	1994	1.7683281	4.15697
Portugal	1995	1.769086	4.1736791
Portugal	1996	1.7670473	4.1877767
Portugal	1997	1.7697096	4.2045669

Portugal	1998	1.7727964	4.2241334
Portugal	1999	1.7745176	4.2390156
Portugal	2000	1.7760988	4.2526438
Portugal	2001	1.7730035	4.2580737
Portugal	2002	1.7753843	4.2590024
Portugal	2003	1.7987282	4.253397
Portugal	2004	1.7939984	4.2590826
Portugal	2005	1.8024142	4.2616299
Portugal	2006	1.7933917	4.2670915
Portugal	2007	1.793401	4.276392
Portugal	2008	1.7904405	4.2757288
Senegal	1990	1.8276604	2.8332209
Senegal	1991	1.8261415	2.8307792
Senegal	1992	1.8254153	2.8228229
Senegal	1993	1.8252824	2.8153933
Senegal	1994	1.8255224	2.8027808
Senegal	1995	1.8259333	2.8135555
Senegal	1996	1.8271751	2.8109497
Senegal	1997	1.8266038	2.8135741
Senegal	1998	1.8261856	2.827989
Senegal	1999	1.8249001	2.8441676
Senegal	2000	1.8234539	2.8470024
Senegal	2001	1.8205345	2.855263
Senegal	2002	1.8185257	2.8466225
Senegal	2003	1.8130725	2.8630281
Senegal	2004	1.8087596	2.8760021
Senegal	2005	1.804553	2.8879136
Senegal	2006	1.799144	2.8866121
Senegal	2007	1.795241	2.8956366
Senegal	2008	1.7919471	2.8993321
Singapore	1990	1.8975848	4.2135738
Singapore	1991	1.9000831	4.2284862
Singapore	1992	1.904206	4.2449507
Singapore	1993	1.9086849	4.2811556
Singapore	1994	1.9212582	4.3112007
Singapore	1995	1.9124905	4.3285144
Singapore	1996	1.9208026	4.3427825
Singapore	1997	1.9347524	4.3636649
Singapore	1998	1.8876562	4.3393787
Singapore	1999	1.9230779	4.3620205
Singapore	2000	1.9465973	4.3920918
Singapore	2001	1.9042909	4.3753374
Singapore	2002	1.9036408	4.3892433
Singapore	2003	1.9196847	4.4151047
Singapore	2004	1.9284179	4.4477211
Singapore	2005	1.9591527	4.4683974
Singapore	2006	1.9701505	4.4907285

Singapore	2007	1.9714973	4.5101435
Singapore	2008	1.9669278	4.4945566
South Africa	1990	1.8548487	3.6862361
South Africa	1991	1.8576287	3.6728388
South Africa	1992	1.8523968	3.6543955
South Africa	1993	1.8536761	3.650549
South Africa	1994	1.8585699	3.6550914
South Africa	1995	1.8600974	3.6590258
South Africa	1996	1.8629845	3.6676742
South Africa	1997	1.8707513	3.6690821
South Africa	1998	1.8769524	3.6611125
South Africa	1999	1.8909943	3.6607506
South Africa	2000	1.8979511	3.6676713
South Africa	2001	1.9024808	3.6705037
South Africa	2002	1.9076342	3.67754
South Africa	2003	1.9195909	3.6845062
South Africa	2004	1.931339	3.6981761
South Africa	2005	1.9332713	3.7148199
South Africa	2006	1.9326228	3.7327917
South Africa	2007	1.9282607	3.750516
South Africa	2008	1.9215288	3.7602259
Spain	1990	1.769727	4.2743179
Spain	1991	1.7706275	4.2842471
Spain	1992	1.7683832	4.286831
Spain	1993	1.75938	4.2809778
Spain	1994	1.7598711	4.2900381
Spain	1995	1.7634346	4.3008355
Spain	1996	1.759797	4.3103467
Spain	1997	1.7579536	4.3257302
Spain	1998	1.7615991	4.3431942
Spain	1999	1.7641156	4.3610418
Spain	2000	1.76689	4.3787781
Spain	2001	1.7647602	4.3891457
Spain	2002	1.7678841	4.3936195
Spain	2003	1.7683883	4.3989797
Spain	2004	1.7728886	4.4054153
Spain	2005	1.7737738	4.4133697
Spain	2006	1.7725752	4.4233801
Spain	2007	1.7727933	4.430194
Spain	2008	1.7680624	4.427121
Sri Lanka	1990	1.7418561	2.8513366
Sri Lanka	1991	1.7416151	2.8644834
Sri Lanka	1992	1.7421241	2.879203
Sri Lanka	1993	1.7427711	2.9027322
Sri Lanka	1994	1.743799	2.9204078
Sri Lanka	1995	1.7440159	2.9377533
Sri Lanka	1996	1.7446621	2.9491876

Sri Lanka	1997	1.7438742	2.9706908
Sri Lanka	1998	1.7421063	2.9856079
Sri Lanka	1999	1.7400371	2.9976509
Sri Lanka	2000	1.7364769	3.0219097
Sri Lanka	2001	1.7323493	3.0221361
Sri Lanka	2002	1.7295217	3.0361663
Sri Lanka	2003	1.7261065	3.0554814
Sri Lanka	2004	1.7248546	3.0726131
Sri Lanka	2005	1.7243644	3.094263
Sri Lanka	2006	1.723829	3.1216452
Sri Lanka	2007	1.7242826	3.146263
Sri Lanka	2008	1.7247903	3.1675237
Sweden	1990	1.798229	4.4889416
Sweden	1991	1.8004088	4.4809756
Sweden	1992	1.7992051	4.4733673
Sweden	1993	1.7845047	4.4617799
Sweden	1994	1.7861937	4.4757794
Sweden	1995	1.7747019	4.4902772
Sweden	1996	1.7834473	4.496532
Sweden	1997	1.7737713	4.5078894
Sweden	1998	1.7745888	4.5255366
Sweden	1999	1.7636069	4.5449783
Sweden	2000	1.7667959	4.5631985
Sweden	2001	1.760387	4.5674803
Sweden	2002	1.7674774	4.5767206
Sweden	2003	1.7663906	4.5851318
Sweden	2004	1.7669116	4.6014367
Sweden	2005	1.7643887	4.6132144
Sweden	2006	1.7654254	4.6290441
Sweden	2007	1.7627892	4.6399838
Sweden	2008	1.7602461	4.6339283
Switzerland	1990	1.8355127	4.6827993
Switzerland	1991	1.8338591	4.6733757
Switzerland	1992	1.8312415	4.6683975
Switzerland	1993	1.8221952	4.6638948
Switzerland	1994	1.8165827	4.6659126
Switzerland	1995	1.8169722	4.6650938
Switzerland	1996	1.8306807	4.6652756
Switzerland	1997	1.82022	4.6730094
Switzerland	1998	1.8450948	4.6834339
Switzerland	1999	1.8156242	4.6873839
Switzerland	2000	1.8293609	4.7006015
Switzerland	2001	1.8165294	4.703219
Switzerland	2002	1.8002168	4.700738
Switzerland	2003	1.8092322	4.6976075
Switzerland	2004	1.8094798	4.7050116
Switzerland	2005	1.8073483	4.7137786

Switzerland	2006	1.8058205	4.7270478
Switzerland	2007	1.8017151	4.7395551
Switzerland	2008	1.8032108	4.7433359
Tanzania	1990	1.8782135	2.4783206
Tanzania	1991	1.881002	2.4729642
Tanzania	1992	1.8837106	2.4609511
Tanzania	1993	1.8862718	2.4517429
Tanzania	1994	1.8881677	2.4447091
Tanzania	1995	1.8899375	2.4470625
Tanzania	1996	1.8901137	2.4544312
Tanzania	1997	1.8894891	2.4582864
Tanzania	1998	1.8881258	2.4633705
Tanzania	1999	1.8859293	2.4732658
Tanzania	2000	1.8826429	2.4833866
Tanzania	2001	1.8782444	2.4976854
Tanzania	2002	1.8727045	2.5165681
Tanzania	2003	1.86633	2.5340891
Tanzania	2004	1.8590219	2.5551387
Tanzania	2005	1.8514051	2.5740304
Tanzania	2006	1.8430715	2.5900408
Tanzania	2007	1.8344293	2.6074318
Tanzania	2008	1.8259587	2.6257653
Thailand	1990	1.7565628	3.1963347
Thailand	1991	1.7585586	3.2274044
Thailand	1992	1.7595429	3.2575582
Thailand	1993	1.7625478	3.2889235
Thailand	1994	1.7649489	3.3231578
Thailand	1995	1.768135	3.3578857
Thailand	1996	1.7697063	3.3785508
Thailand	1997	1.7664513	3.3678775
Thailand	1998	1.7572614	3.3146597
Thailand	1999	1.7602523	3.3284535
Thailand	2000	1.7600859	3.3435613
Thailand	2001	1.7599696	3.347847
Thailand	2002	1.7586916	3.3653583
Thailand	2003	1.76009	3.3906346
Thailand	2004	1.762157	3.4133321
Thailand	2005	1.7618224	3.4297447
Thailand	2006	1.7584415	3.4491739
Thailand	2007	1.7573945	3.4692764
Thailand	2008	1.7551087	3.4792217
Tunisia	1990	1.7530764	3.3015166
Tunisia	1991	1.7535961	3.3095138
Tunisia	1992	1.7511166	3.3332824
Tunisia	1993	1.7521056	3.3342052
Tunisia	1994	1.7486095	3.3399395
Tunisia	1995	1.7472014	3.3430896

Tunisia	1996	1.7465464	3.3667223
Tunisia	1997	1.7435838	3.3837667
Tunisia	1998	1.7426493	3.3985208
Tunisia	1999	1.7404936	3.4183828
Tunisia	2000	1.7415266	3.4334564
Tunisia	2001	1.7396841	3.4492969
Tunisia	2002	1.7392324	3.452227
Tunisia	2003	1.739134	3.4731314
Tunisia	2004	1.7383576	3.4948037
Tunisia	2005	1.7364198	3.5077157
Tunisia	2006	1.734637	3.5260882
Tunisia	2007	1.7322294	3.5486555
Tunisia	2008	1.7321678	3.5638682
Turkey	1990	1.8140872	3.7000886
Turkey	1991	1.8113252	3.6958947
Turkey	1992	1.8072356	3.7101402
Turkey	1993	1.8067183	3.7352285
Turkey	1994	1.7928489	3.7076259
Turkey	1995	1.794331	3.7337621
Turkey	1996	1.7874006	3.7579086
Turkey	1997	1.7851325	3.7828893
Turkey	1998	1.781537	3.7861197
Turkey	1999	1.7763387	3.7646753
Turkey	2000	1.778184	3.7866969
Turkey	2001	1.7650983	3.7549018
Turkey	2002	1.7654075	3.7746762
Turkey	2003	1.764678	3.7908861
Turkey	2004	1.7646815	3.8238202
Turkey	2005	1.7659403	3.8530642
Turkey	2006	1.7657659	3.8763894
Turkey	2007	1.7658978	3.8907336
Turkey	2008	1.7634703	3.888201
Uganda	1990	1.9037982	2.2958739
Uganda	1991	1.9078915	2.3042283
Uganda	1992	1.9117267	2.3039577
Uganda	1993	1.9149769	2.3240992
Uganda	1994	1.9173263	2.3367501
Uganda	1995	1.9184346	2.3700802
Uganda	1996	1.9174133	2.394048
Uganda	1997	1.914957	2.4021325
Uganda	1998	1.9111551	2.4094556
Uganda	1999	1.9056564	2.4294465
Uganda	2000	1.8986239	2.428923
Uganda	2001	1.8910093	2.4365753
Uganda	2002	1.8827259	2.4583745
Uganda	2003	1.8744929	2.4708855
Uganda	2004	1.8662164	2.4847145

Uganda	2005	1.8583699	2.4966515
Uganda	2006	1.8509739	2.5264665
Uganda	2007	1.8435234	2.5469238
Uganda	2008	1.8364251	2.5685956
United Kingdom	1990	1.8058698	4.4138754
United Kingdom	1991	1.8077035	4.4068828
United Kingdom	1992	1.8040761	4.4112951
United Kingdom	1993	1.8060463	4.4251544
United Kingdom	1994	1.8015763	4.4450453
United Kingdom	1995	1.7999733	4.4589768
United Kingdom	1996	1.8026092	4.4727749
United Kingdom	1997	1.7995585	4.4901506
United Kingdom	1998	1.8045739	4.5041065
United Kingdom	1999	1.7977027	4.5152358
United Kingdom	2000	1.7962904	4.5322276
United Kingdom	2001	1.7962382	4.5400284
United Kingdom	2002	1.7949004	4.5480207
United Kingdom	2003	1.7962514	4.5628285
United Kingdom	2004	1.7985286	4.5739218
United Kingdom	2005	1.7948737	4.5847918
United Kingdom	2006	1.7960689	4.593439
United Kingdom	2007	1.7953083	4.6047418
United Kingdom	2008	1.7928264	4.5980208
United States of America	1990	1.8854922	4.5180582
United States of America	1991	1.8825949	4.5119392
United States of America	1992	1.8794476	4.5210881
United States of America	1993	1.8878996	4.527122
United States of America	1994	1.8885399	4.5389819
United States of America	1995	1.8851261	4.5454595
United States of America	1996	1.8839396	4.5565869
United States of America	1997	1.884363	4.5704222
United States of America	1998	1.885224	4.5842673
United States of America	1999	1.8871809	4.5998342
United States of America	2000	1.8929952	4.6124132
United States of America	2001	1.8878639	4.6122154
United States of America	2002	1.8891188	4.6158322
United States of America	2003	1.8843251	4.6240543
United States of America	2004	1.8867551	4.6362241
United States of America	2005	1.8856531	4.6465369
United States of America	2006	1.8806988	4.6537782
United States of America	2007	1.8797806	4.6573526
United States of America	2008	1.8734257	4.6519818
Uruguay	1990	1.7352527	3.5976509
Uruguay	1991	1.7359492	3.6096933
Uruguay	1992	1.7370085	3.6397026
Uruguay	1993	1.7371336	3.6479099
Uruguay	1994	1.7355076	3.6752523

Uruguay	1995	1.7337284	3.6657904
Uruguay	1996	1.7366033	3.6862498
Uruguay	1997	1.7379053	3.7188102
Uruguay	1998	1.7374458	3.7352085
Uruguay	1999	1.7401942	3.7244395
Uruguay	2000	1.7342431	3.7144095
Uruguay	2001	1.7308965	3.6966116
Uruguay	2002	1.7248531	3.6615663
Uruguay	2003	1.7240242	3.6653224
Uruguay	2004	1.7265277	3.6867365
Uruguay	2005	1.7272116	3.7178097
Uruguay	2006	1.7298417	3.7345938
Uruguay	2007	1.7296702	3.7610489
Uruguay	2008	1.7294636	3.7897814
Venezuela	1990	1.7803923	3.7229845
Venezuela	1991	1.7802333	3.7529182
Venezuela	1992	1.7766761	3.7684325
Venezuela	1993	1.7804089	3.7599007
Venezuela	1994	1.7784478	3.7401078
Venezuela	1995	1.7801979	3.7476898
Venezuela	1996	1.7701694	3.7377894
Venezuela	1997	1.7757924	3.7557817
Venezuela	1998	1.7875409	3.7484177
Venezuela	1999	1.7738395	3.7132152
Venezuela	2000	1.7608793	3.7206299
Venezuela	2001	1.7734358	3.726967
Venezuela	2002	1.7611536	3.6786829
Venezuela	2003	1.7672492	3.6357488
Venezuela	2004	1.7563919	3.7009406
Venezuela	2005	1.7580362	3.735971
Venezuela	2006	1.7641216	3.7693676
Venezuela	2007	1.769079	3.7984461
Venezuela	2008	1.7686329	3.8135513
Vietnam	1990	1.735134	2.479017
Vietnam	1991	1.7329785	2.496173
Vietnam	1992	1.7309951	2.5244561
Vietnam	1993	1.7295384	2.5506595
Vietnam	1994	1.7280378	2.5801472
Vietnam	1995	1.72687	2.6126
Vietnam	1996	1.7257859	2.6444304
Vietnam	1997	1.7249768	2.6716903
Vietnam	1998	1.7234028	2.6893637
Vietnam	1999	1.7210328	2.7031009
Vietnam	2000	1.720194	2.725796
Vietnam	2001	1.7192982	2.7463875
Vietnam	2002	1.7193602	2.7679659
Vietnam	2003	1.720642	2.791893

Vietnam	2004	1.7219198	2.8182484
Vietnam	2005	1.7213951	2.8447802
Vietnam	2006	1.7213451	2.8692547
Vietnam	2007	1.7211372	2.8944556
Vietnam	2008	1.7206651	2.9137511
Zambia	1990	1.9436533	2.830481
Zambia	1991	1.9512386	2.8197295
Zambia	1992	1.9581309	2.8019347
Zambia	1993	1.9642861	2.8203975
Zambia	1994	1.9677591	2.7709094
Zambia	1995	1.9713299	2.7477456
Zambia	1996	1.9729315	2.765661
Zambia	1997	1.9740651	2.7681021
Zambia	1998	1.9720958	2.7481226
Zambia	1999	1.9679535	2.7459696
Zambia	2000	1.9632252	2.7495764
Zambia	2001	1.9555898	2.7592432
Zambia	2002	1.9461531	2.76252
Zambia	2003	1.9350322	2.7733745
Zambia	2004	1.9233811	2.7852042
Zambia	2005	1.9107634	2.7964729
Zambia	2006	1.8980064	2.811147
Zambia	2007	1.8845522	2.8251936
Zambia	2008	1.871981	2.8384303
Zimbabwe	1990	1.8250779	2.8315834
Zimbabwe	1991	1.8333098	2.8438493
Zimbabwe	1992	1.8439558	2.792887
Zimbabwe	1993	1.8549061	2.7884454
Zimbabwe	1994	1.8703908	2.8184495
Zimbabwe	1995	1.8832677	2.8112011
Zimbabwe	1996	1.8982584	2.8463691
Zimbabwe	1997	1.913086	2.8505971
Zimbabwe	1998	1.9256964	2.8563775
Zimbabwe	1999	1.9398646	2.8473334
Zimbabwe	2000	1.9492919	2.8296896
Zimbabwe	2001	1.9569775	2.83302
Zimbabwe	2002	1.9618813	2.7907022
Zimbabwe	2003	1.9599328	2.7087018
Zimbabwe	2004	1.9537464	2.682035
Zimbabwe	2005	1.9469848	2.6558959
Zimbabwe	2006	1.9349246	2.640128
Zimbabwe	2007	1.9207257	2.623424
Zimbabwe	2008	1.9013944	2.5374944
